# A Firefly Luciferase Dual Color Bioluminescence Reporter Assay Using Two Substrates To Simultaneously Monitor Two Gene Expression Events

**DOI:** 10.1038/s41598-018-24278-2

**Published:** 2018-04-16

**Authors:** Bruce R. Branchini, Tara L. Southworth, Danielle M. Fontaine, Dawn Kohrt, Catherine M. Florentine, Martha J. Grossel

**Affiliations:** 1 Department of Chemistry, Connecticut College, New London, Connecticut 06320 USA; 2 Department of Biology, Connecticut College, New London, Connecticut 06320 USA

## Abstract

Effective methods for monitoring eukaryotic gene expression and regulation based on bioluminescence - the emission of light by living organisms - are well established. Typically, the expression of a gene of interest is reported on with high sensitivity and over a wide dynamic range by the emission of light from a variety of engineered luciferase genes from beetles and marine organisms. The luciferase reporter genes are expressed downstream of the target gene or promoter and detected after exogenous addition of luciferin substrates. We describe a novel bioluminescence reporter method for the simultaneous monitoring of two genes expressing engineered firefly luciferase variants that emit readily distinguishable green and red light signals. The key feature is the selectivity of the enzymes for two luciferin substrates that determine each emission color. To validate our method, we performed a complex promoter transactivation experiment side-by-side with the Dual-Luciferase Reporter protocol and obtained essentially identical results. Additional comparative experiments demonstrated that our assay system provided improvements in background, cell normalization, and detectability compared to representative available methods. With access to a luminometer equipped with two optical filters, this method is an excellent choice for genetic reporter assays that can be performed with a single reagent solution.

## Introduction

Effective methods for monitoring gene expression and regulation based on bioluminescence (BL) – the emission of light by living organisms – are well known^1–3^. The expression of a gene of interest is reported on with high sensitivity and over a wide dynamic range by the emission of light from a variety of engineered luciferase (Luc) genes from beetles and marine organisms that are expressed downstream of the target gene after exogenous addition of their respective luciferin substrates. While reporter assays are typically conducted on lysates from cultured human cells, BL detection also enables *in vivo* cell and animal imaging and biosensor applications as recently reviewed^1–3^. When performing reporter assays, especially when using transiently transfected cells, it is essential to normalize the results because controls are needed to account for cell number, viability, and transfection efficiency^4^. Normalization has been achieved in dual-reporter assays that monitor control, *e*.*g*. cytomegalovirus (CMV) or thymidine kinase (TK) promoters, and test gene activities using two BL signals. Perhaps the most popular format, typified by the Promega Dual-Luciferase^®^ Reporter (DLR^®^) assay system^5^, involves two unrelated luciferases that are assayed sequentially without filters using two substrates and a Luc inhibitor. Specifically, the DLR^®^ system uses firefly (*Photinus pyralis*) and sea pansy (*Renilla reniformis*) enzymes with their respective substrates firefly luciferin (LH_2_) plus ATP and coelenterazine. A newer version of the DLR^®^ system called NanoDLR™ ^6^, uses the NanoLuc and firefly enzymes along with substrates furimazine and 5-fluoroluciferin. The method offers improvements that may provide 2- to 100-fold greater sensitivity^6^ than DLR^®^. While several laboratories have conducted studies^7,8^ with NanoDLR™, we were unable to find comparative data on this new technology. An interesting application of the non homologous enzyme – two substrate sequential format is the use of secreted NanoLuc^®^ ^9^, and *Gaussia* enzymes eliminating the need for cell lysis^10^. Cross reactivity of the enzymes, which share only 22% sequence identity, with the furimazine and coelenterazine substrates, however, limits the ease of performance. This is because in transfection experiments with plasmids under CMV control, a significant signal from NanoLuc^®^, equivalent to ~25% of the Gaussia response, was observed with the coelenterazine-containing Gaussia assay reagent^10^. In turn, this necessitated an extensive series of control experiments so that a standard curve could be constructed to determine the contribution of NanoLuc^®^ activity to the signal obtained with the coelenterazine reagent^10^. The cross-reactivity is ~3-fold higher than expected as estimated from the reported^9^ ~30-fold greater luminescence of NanoLuc^®^ with furimazine over coelenterazine.

A drawback of using two dissimilar luciferases as is the case with the DLR^®^ systems, especially in the screening of libraries of chemical compounds, is that selective inhibition of the signals may occur leading to misinterpretation of the data^11^. This problem can be minimized by using a second type of reporter system like the Chroma-Glo™ method^12^ that employs highly similar (~99%) red- and green-emitting click beetle Lucs CBR and CBG99 with a single substrate (LH_2_). A similar strategy has been employed with click beetle and railroad worm Lucs (~46% DNA similarity) that use LH_2_ for dual-color BL imaging^13^. In the Chroma-Glo™ system, the two signals are produced simultaneously, but with significant spectral overlap that necessitates the use of transmission filters to separate them. It is likely that this assay system has not been widely adopted because of lower signal sensitivity (~10-fold compared to the DLR^®^ method) and requirements for extensive controls needed for cumbersome calculations to determine the intensities of each signal.

**Figure 1:**
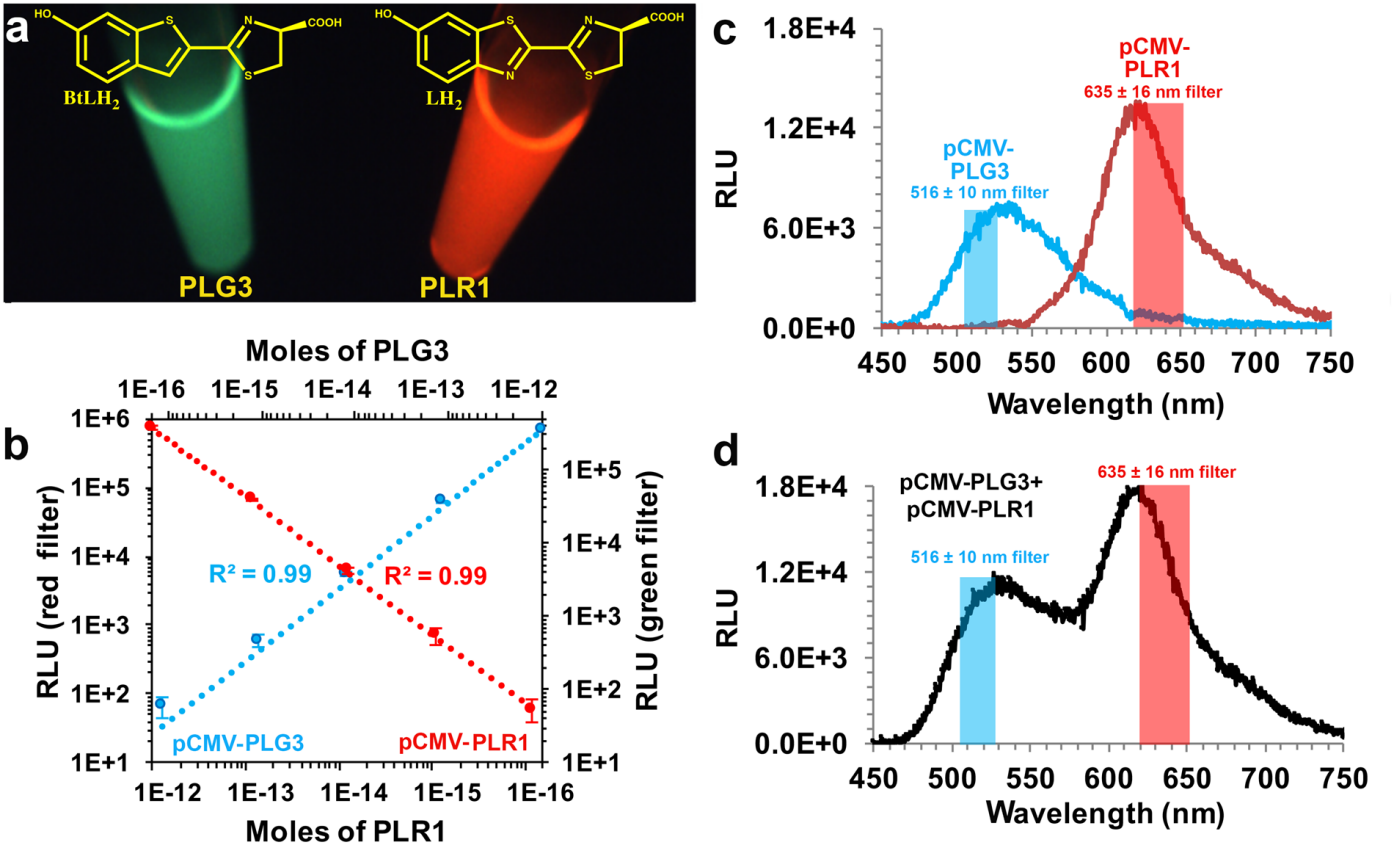
Bioluminescence emission and detectability of PLG3 and PLR1 assayed with DSM. (**a**) Photograph of *in vitro* BL reactions of 1 µg PLG3 and PLR1 with DSM. Chemical structures of the benzothiophene (BtLH_2_) and firefly (LH_2_) luciferins responsible for each emission color are shown. (**b)** Simultaneous detection of mixtures of lysates from equal numbers of HEK293T cells transfected with pCMV-PLG3 or pCMV-PLR1 using a Synergy™ 2 microplate reader equipped with narrow bandpass filters. The relative light units (RLU) measured through the 516 ± 10 nm filter (blue line) and 635 ± 16 nm filter (red line) represent the activity expressed from the pCMV-PLG3 and pCMV-PLR1 plasmids, respectively. (**c**–**d**) BL emission spectra of lysates from equal numbers of HEK293T cells transformed with pCMV-PLG3 or pCMV-PLR1. The lysates were assayed (**c**) individually or (**d**) mixed together.

We have developed a new dual-color assay format called DART (**D**ual **A**nalyte **R**eporter with **T**wo firefly luciferase substrates) that combines several advantages of the current method types, while minimizing the drawbacks of each. BL signals are produced from lysates containing two highly similar (99%) *P*. *pyralis* variants upon mixing with a single solution (DSM, dual substrate mix) containing substrates LH_2_, benzothiophene luciferin (BtLH_2_)^14^ (Fig. 1a), ATP, MgSO_4_, and DTT. A key feature of the two homologous enzyme – two substrate DART method is the selectivity of the Lucs for the substrates that produce very well separated emission spectra.

## Results and Discussion

### Rationale for construction of pCMV-PLR1 and pCMV-PLG3

Starting with PLG2^15^, a thermostable and pH resistant (to emission color shifting) chimeric Luc that emits with λ_max_ = 559 nm in HEK293T cell lysates, we used random and rational mutagenesis techniques to construct green (PLG3)- and red (PLR1)-emitting Lucs (Supplementary Fig. 1) with enhanced catalytic efficiencies for BtLH_2_ and LH_2_ (Table 1 and Fig. 1a), respectively. To construct PLR1, we first introduced two residue changes into PLG2 that we previously found had red-shifted the BL emission, Tyr255Phe^16^ and Ser284Thr^17^. Based on the results of several rounds of random mutagenesis, the changes encoding Phe250Thr, Leu291Ile, and Tyr340Phe were introduced into PLG2 to produce a Luc variant gene with a preference for BtLH_2_. In addition, Val351 was changed to Ile in both PLR1 and PLG3 and Phe465 to Arg for increased thermostability in PLG3.

**Table 1:** Steady-state kinetics of purified Luc proteins at pH 7.8.

Enzyme	*k*_cat_ (s^−1^, x10^−3^)		*K*_m_ (µM)		*k*_cat/_*K*_m_ (mM^−1^s^−1^)		LH_2_:BtLH_2_ (*k*_cat/_*K*_m_)
	LH_2_	BtLH_2_	LH_2_	BtLH_2_	LH_2_	BtLH_2_	
PpyWT	27 ± 3	2.8 ± 0.8	15 ± 2	6 ± 1	1.8	0.47	3.8
PLR1	12 ± 1	0.20 ± 0.03	37 ± 4	28 ± 3	0.32	0.007	45.7
PLG3	4.7 ± 0.3	2.3 ± 0.1	15 ± 3	5 ± 1	0.31	0.46	0.67

### Bioluminescence properties of PLR1 and PLG3

Using purified proteins, emission maxima of 518 nm and 620 nm were realized with the PLG3-BtLH_2_ and PLR1-LH_2_ combinations (Supplementary Table 1 and Supplementary Fig. 2). With DSM, which contains both substrates, lysates of HEK293T cells transfected individually with pCMV-PLG3 or pCMV-PLR1 produced distinct emission profiles with λ_max_ values of 528 nm and 620 nm when assayed alone or mixed together (Fig. 1c,d). The well-separated emission spectra were achieved in large part due to the relative preferences of PLG3 for BtLH_2_ (1.5-fold) and PLR1 for LH_2_ (46-fold). With this high degree of signal separation (Fig. 1c), narrow bandpass filters were used to simultaneously measure (Fig. 1b) green and red signals providing very minimal spectral overlap (Fig. 1c).

### Detectability of pCMV-PLR1 and pCMV-PLG3 in HEK293T cell lysates

To determine the detectability of PLG3 and PLR1 in the presence of each other, the proteins were expressed individually under the control of the CMV promoter at 37 °C in HEK293T cells. Lysates of equivalent cell count were prepared and mixtures containing both enzymes were assayed with DSM using a microplate luminometer and a single injector. The simultaneously monitored green and red signals could be measured at levels corresponding to 0.2 fmol to 2.0 pmol (Fig. 1b). Under the CMV promoter, the red signal is ~2-fold stronger and contributes only ~0.6% of its intensity to the green luminescence (Fig. 1c). Therefore, optimal results will be obtained using the red signal as the genetic reporter and the green signal as the normalization control.

### Comparison of DART and Chroma-Glo™ Methods

**Figure 2:**
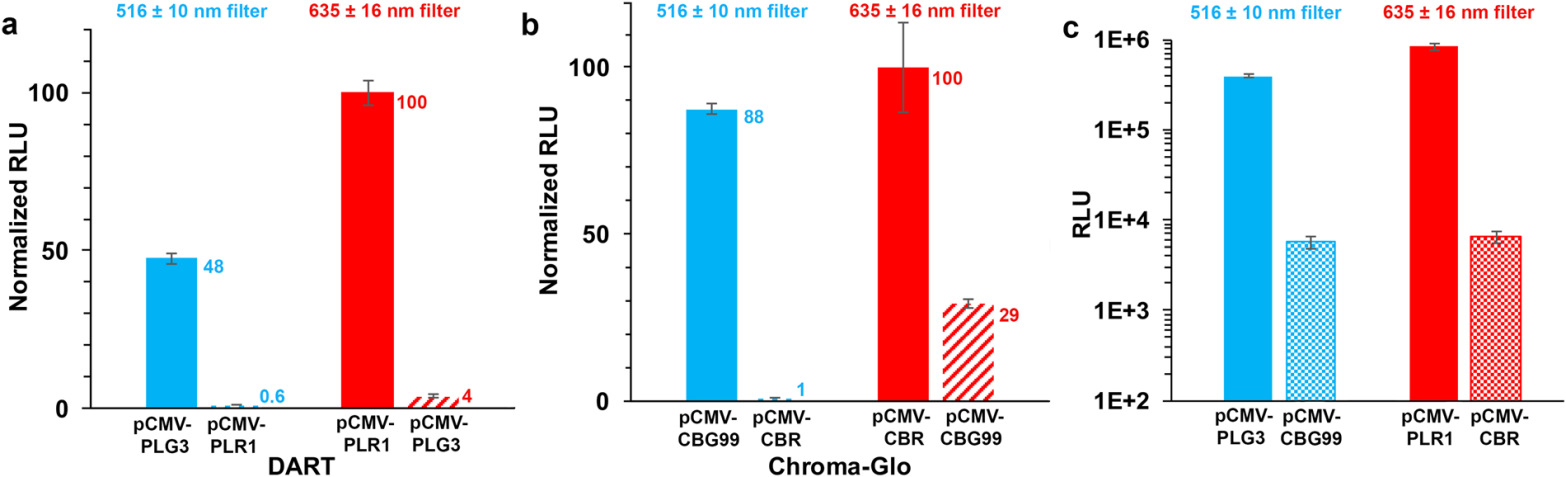
Comparison of the DART and Chroma-Glo™ Luciferase assay systems. Normalized spectral overlap measured with **(a)** DART and **(b)** Chroma-Glo™ using lysates from equal numbers of HEK293T cells transformed with **(a)** pCMV-PLG3 and pCMV-PLR1 assayed with DSM or **(b)** pCMV-CBG99 and pCMV-CBR assayed with Chroma-Glo™ reagent. Assays were performed using a Synergy™ 2 microplate luminometer equipped with the indicated filters. The relative emission from pCMV-PLR1 or pCMV-CBR detected through the 516 ± 10 nm filter (blue hatched bars) and the relative emission from pCMV-PLG3 or pCMV-CBG99 detected through the 635 ± 16 nm filter (red hatched bars) are shown. (**c**) BL emission from the same experiments detected through the indicated filters. The mean relative light units (RLU) ± STD for triplicate assays are depicted.

We first compared the relative intensities and spectral overlap produced by the enzymes in the DART and Chroma-Glo™ systems with their respective reagents. While both methods offer the advantages of using two very similar genes/Lucs (from beetles) and simultaneous detection of green and red signals, the Chroma-Glo™ system requires only a single LH_2_ substrate and a reagent that produces optimal BL with the click beetle Lucs. In parallel experiments using identically prepared HEK293T cell lysates containing the DART and Chroma-Glo™ Lucs, the BL of individual Lucs was measured with both methods using a set of green and red filters (Fig. 2). While negligible overlap of the red signals was detected (measured through the green filter) with either method (Fig. 2a,b), ~30% spectral overlap of the green signal was monitored through the red filter with the Chroma-Glo™ method, compared to ≤4% overlap with the DART method (Fig. 2a,b). The high degree of spectral overlap of the Luc signals in the Chroma-Glo™ method necessitates the use of a “calculator” and many time- and reagent-consuming controls in order to extract the red and green signals (Fig. 2b)^12^. With the DART method, however, the very low overlap of both signals through filters (Fig. 2a) renders it unnecessary to mathematically extract each signal over a wide concentration range of the analytes. This is because the spectral overlap is approximately equal to the standard deviation of the signal intensity measurements when equal amounts of the Lucs are present. In practice, using PLG3 as the normalization control at a conservative 5 times the background, red signals can be measured over 3 orders of magnitude without increasing the green signal above the error of the measurement. By increasing the amount of the control plasmid, the dynamic range would remain approximately the same, but higher levels of red signal could be measured as necessary. We also examined the relative intensities of the BL signals emitted by the Lucs used in each method and determined that the intensities of the red- and green-emitting DART Lucs were 130-fold and 70-fold greater than those of the corresponding click beetle enzymes (Fig. 2c). These results likely reflect inherent lower specific activity and possibly reduced expression of the click beetle Lucs.

### Validation of DART method

**Figure 3:**
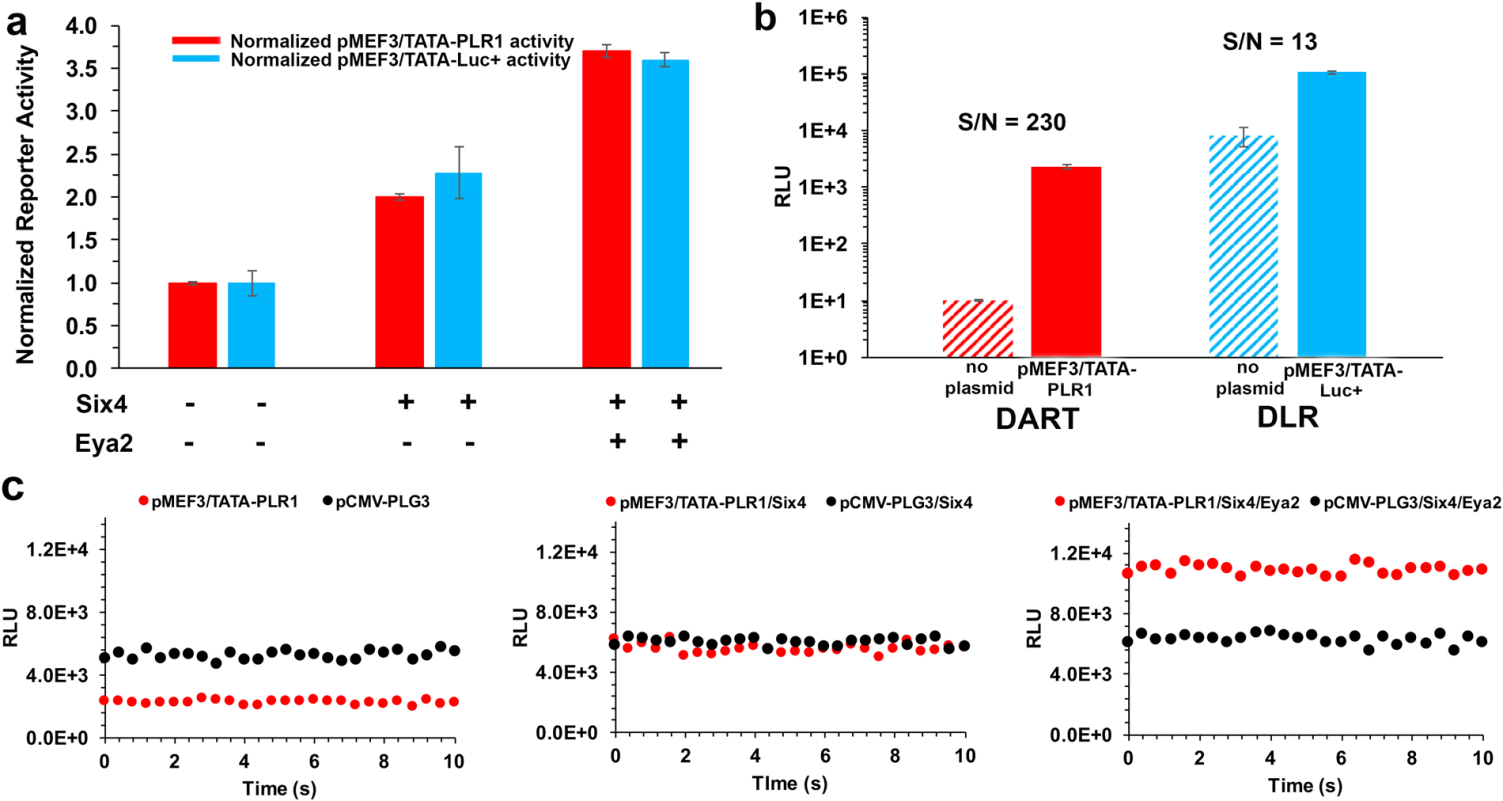
Comparison of the DART and DLR^®^ reporter methods. In separate experiments, HEK293T cells were co-transfected with pMEF3/TATA-PLR1 or pMEF3/TATA-Luc+ in the presence and absence of plasmids expressing Six4 and Eya2. The pCMV-PLG3 or pRL-CMV Lucs were included as the corresponding normalization controls. (**a**) After normalization to the pCMV-PLG3 or pRL-CMV activity, the fold reporter gene activation of pMEF3/TATA-PLR1 or pMEF3/TATA-Luc+ in the absence of both Six4 and Eya2 was set to 1.0. All reporter signals were expressed as the mean fold-amplification ± STD (duplicate transfections, assayed in triplicate). The normalized reporter activities were not significantly different (*t*-test) between the two methods when comparing MEF3/TATA alone (*P* = 0.97), in the presence of Six4 (*P* = 0.14), and in the presence of Six4 and Eya2 (*P* = 0.22). (**b**) The S/N of the reporter methods is represented by the ratio of the raw signals from pMEF3/TATA-PLR1 and pMEF3/TATA-Luc+ compared to those of the corresponding lysed untransfected cells (hatched bars). (**c**) Plots showing the simultaneous monitoring of the pCMV-PLG3 normalization control (black) and pMEF3/TATA-PLR1 reporter (red) signals using DART (with filters) to produce the normalized data illustrated with red bars in panel a.

To validate the DART method, we performed a previously described reporter experiment^18,19^ that employed the widely used DLR^®^ system. In HeLa^18^ and Cos-7^19^ cells, the transactivation of the myocyte expression factor-3 promoter (MEF3/TATA) by Six4 (a sine oculis protein) and Eya2 (a human homologue of the eyes absent protein) was evaluated. With DART, the activation of MEF3/TATA in HEK293T cells revealed a 2-fold increase in transcription when the transcriptional activating protein Six4 was expressed. This increased to 3.5-fold with the co-expression of the Eya2 protein (Fig. 3a). These results were essentially identical (*t*-test) when comparing MEF3/TATA alone (*P* = 0.97), in the presence of Six4 (*P* = 0.14), and in the presence of Six4/Eya2 (*P* = 0.22) with those obtained using the DLR^®^ system. Interestingly, DART provided ~18-fold greater S/N for the MEF3/TATA reporter signal even though the corresponding DLR^®^ measurement was ~45-fold higher (Fig. 3b). The greater S/N of the DART method results from the lower background produced by DSM and the use of transmission filters. In Fig. 3c, we show the raw data obtained by simultaneously monitoring the PLG3 normalization control and PLR1 reporter signals used to produce the DART results presented in Fig. 3a. In contrast, the data (Fig. 3a) obtained using the DLR^®^ system were necessarily acquired in a discontinuous manner.

## Conclusion

The novel DART format, which features two substrates and two highly similar firefly Luc enzymes, provides a straightforward and robust method for reporter assays of cell lysates. The impressive signal separation eliminates the need to do extensive controls and calculations to manage poor signal overlap. The low cost is mainly attributable to the use of DSM, a simple single assay solution that does not contain expensive additives. The dynamic range of the method spans at least 3 orders of magnitude with exceptionally low background noise. The S/N advantage of DART was mainly achieved by the reduction of the background signal with narrow bandpass filters. While the filters also attenuated the signals by ~80%, the specific activity of the enzymes was sufficiently high to readily enable measurements at the femtomol level. Additional major features of DART are compared to the established reporter formats typified by the Promega DLR^®^ and Chroma-Glo™ methods (Supplementary note).

The stable glow kinetics characterized by signal half-lives of ~3 h could make our method amenable to high throughput screening^2,20^ and to performing assays without injectors (Supplementary Fig. 3). The DART method, however, does require the use of filters and the BtLH_2_ substrate is not yet commercially available, but can be obtained from the authors. We are presently investigating the feasibility of extending the principle of the DART method to *in vivo* imaging applications.

## Materials and Methods

### Materials

The following materials were obtained from the sources indicated: Mg-ATP (bacterial source) and ATP (disodium salt hydrate) from Sigma-Aldrich (St. Louis, MO); and DLR Assay System, pRL-CMV, expressing *Renilla* luciferase driven by a CMV promoter, and passive lysis buffer (PLB) from Promega (Madison, WI). Firefly luciferin potassium salt, Chroma-Glo™ reagent, the pF4Ag plasmid (non-commercial expression vector driven by a CMV promoter), pCBG99-Basic and pCBR-Basic vectors, and pGL4.54 (containing *luc2* driven by the TK promoter) were generous gifts from Promega (Madison, WI). The pMEF3/TATA-Luc + plasmid, containing six MEF3 sites and a TATA sequence preceding the *luc*+ gene in the Promega pGL3 Basic vector; the pcDNA3.1-Flag-Eya2 plasmid expressing Eya2; and pcDNA3.1-Six4 expressing Six4 were kind gifts from the Pascal and Casey labs^18,21^. Benzothiophene luciferin was prepared as previously described^14^ and is available upon request. DSM was prepared by diluting stock solutions of the reagents to the final concentrations indicated in 50 mM Tricine, pH 7.8: 0.48 mM ATP, 1.8 mM MgSO_4_, 18 mM DTT, 0.12 mM LH_2_ and 0.24 mM BtLH_2_.

### Construction of pCMV-PLR1 and pCMV-PLG3

Site-directed mutagenesis to construct pCMV-PLR1 was performed by introducing the Tyr255Phe and Ser284Thr changes and reverting Val351 to Ile (Supplementary Fig. 1) with the QuikChange® Lightning Site-Directed Mutagenesis kit (Agilent, Santa Clara, CA) using human codon optimized PLG2 in the pGEX-6P-2 plasmid^15^ as the template. The PLR1 cDNA was then sublconed into the pF4Ag plasmid, which contains a CMV promoter. Plasmid pCMV-PLG3 was constructed by screening an in-house library of approximately 75 *P*. *pyralis* variants and identifying the Phe250Thr mutation, which produced the optimal combination of ~2-fold improved substrate specificity (*k*_cat_/*K*_m_) towards BtLH_2_ and blue-shifted emission spectra (λ_max_ = 517 nm). Two rounds of PCR-based random mutagenesis with the GeneMorph® II EZClone Domain Mutagenesis Kit (initially with the *P*. *pyralis* F250T variant in the pGEX-6P-2 plasmid), in which ~4,500 colonies were screened, sequentially identified the Tyr340Phe and Leu291Ile mutations that together improved the BtLH_2_ selectivity an additional 6-fold with a modest 5 nm red shift in emission. The screening process involved visualization of BL in XL-10 Gold cells initially with LH_2_ to identify dim colonies. Selected colonies were streaked in duplicate and screened with either BtLH_2_ or LH_2_ to identify those that were brighter with BtLH_2_ than LH_2_. The three mutations Phe250Thr, Leu291Ile, and Tyr340Phe were then introduced into the human codon optimized PLG2 cDNA in the pGEX-6P-2 plasmid^15^ followed by reverting Val351 to Ile and changing Phe465 to Arg (Supplementary Fig. 1) to match the thermostability of PLG3 at 37 °C to that of PLR1 (~24 h half-life). The PLG3 cDNA was then subcloned into the pF4Ag plasmid, which contains a CMV promoter. While the latter mutations improved the overall thermostability and narrowed the emission spectrum bandwidth, they were also responsible for reducing the substrate specificity for BtLH_2_ by ~2-fold. The sequences of luciferase genes in the pGEX-6P-2 and pF4Ag vectors were verified by DNA sequencing at the W. M. Keck Biotechnology Laboratory (Yale University, New Haven, CT). The constructs in the pF4Ag plasmid are referred to as pCMV-PLG3 and pCMV-PLR1 throughout the manuscript. The pCMV-CBG99 and pCMV-CBR constructs were prepared by replacing the PLG3 and PLR1 cDNA sequences with that of CBG99 and CBR, respectively, in the pF4Ag plasmid. Methods for protein expression in the pGEX-6P-2 vector and purification using the Glutathione Sepharose® 4B affinity chromatography were previously reported^22^.

### Steady-State Kinetic Constants

Values of *K*_m_ and *V*_max_ for LH_2_ and Bt-LH_2_ with purified PLR1 and PLG3 were determined from BL activity assays in which measurements of maximal light intensities (bursts) were taken as estimates of initial velocities. The equipment used to make these measurements has been previously described^23^. Data were collected from 0.525 ml reactions in 50 mM Tricine buffer, pH 7.8, containing 0.6 μg of Luc enzyme in 20 mM Tris-HCl (pH 7.8) containing 150 mM NaCl, 1 mM EDTA, 1 mM DTT, 0.8 M ammonium sulfate and 2% glycerol. The concentrations of LH_2_ and Bt-LH_2_ were varied (1 μM–0.5 mM). Reactions were initiated by injection of Mg-ATP (2 mM final concentration). Kinetic constants (Table 1) were determined using the nonlinear least squares method of the Enzyme Kinetics Pro software (SynTex), which fits data from the Michaelis–Menten equation to a rectangular hyperbola; *k*_cat_ was determined from initial velocities taken as peak light emission 1 min after the initiation of bioluminescence.

### Transfection of HEK293T cells and preparation of lysates

In all experiments described below, HEK293T cells were plated in 24 well plates at a density of 150,000 cells/well in Dulbecco’s Modified Eagle’s Medium (Corning) +10% fetal bovine serum and grown overnight. The cells were transfected in triplicate with varying amounts of each plasmid (transfection mix included 500 ng total DNA including empty vector and 1.5 µL Lipofectamine 2000 in 0.1 mL OptiMEM) and grown overnight at 37 °C with CO_2_. The next day growth media were removed; the cells were washed with PBS, and lysed in 0.1 mL PLB.

### Bioluminescence emission of PLR1 and PLG3 in HEK293T cell lysates

Lysates (5 μL) from HEK293T cells transfected with pCMV-PLR1 or pCMV-PLG3 were added individually or mixed together into cuvettes containing 0.5 mL of DSM with gentle mixing. After 1 min, BL spectra were recorded (Fig. 1c,d) with a Horiba Jobin-Yvon iHR imaging spectrometer equipped with a liquid N_2_ cooled CCD detector. Data were collected at 22 °C over the wavelength range 450 nm–750 nm with the emission slit width set to 5 nm and were corrected for the spectral response of the CCD using a correction curve provided by the manufacturer.

### Detectability of PLR1 and PLG3 in HEK293T cell lysates

Stock solutions of lysates from HEK293T cells transfected with pCMV-PLR1 or pCMV-PLG3 (prepared as described above) were diluted 10-fold in PBS containing 1 mg/ml BSA. Serial 10-fold dilutions of the pCMV-PLR1 and pCMV-PLG3 lysates were made and 5 µL of each dilution of pCMV-PLR1 was mixed with 5 µL of the same dilution of pCMV-PLG3 in the wells of a white 96-well plate. Bioluminescence was initiated by automatic injection of 0.1 mL of DSM. After a 1 min delay, emission signals were recorded on a Synergy™ 2 microplate luminometer (BioTek, Winooski, VT) using 516 ± 10 nm (green) and 635 ± 16 nm (red) filters (BioTek). The instrument records the mean intensity of the flat signals over 10 s by alternately taking readings for 0.02 s with the green and red filters. Data shown in Fig. 1b were expressed in moles of each Luc and were obtained from a standard curve constructed using known amounts of purified proteins.

### Comparison of the DART and Chroma-Glo™ Luciferase assay systems

Equal numbers of HEK293T cells were transfected with the following plasmids: pCMV-PLR1, pCMV-PLG3, pCMV-CBR, or pCMV-CBG99, and cell lysates were prepared as described above. In triplicate experiments, lysates from each plasmid were diluted 10-fold in PBS and 5 µL of each dilution were added to the wells of a white 96-well plates. For pCMV-PLR1 and pCMV-PLG3, DART assays were performed as described below. The BL of pCMV-CBR and pCMV-CBG99 were assayed according to the manufacturer’s protocol^12^ using the Chroma-Glo™ reagent except that cells were lysed with PLB and the Biotek filters were used.

### The DART assay

To perform the DART assay, HEK293T cells were plated in 24 well plates at a density of 150,000 cells/well in Dulbecco’s Modified Eagle’s Medium (Corning) +10% fetal bovine serum. The next day cells were co-transfected in duplicate with plasmids expressing PLR1 (reporter) under control of the promoter of interest and PLG3 (normalization control) under control of a CMV or TK promoter. The transfection mix included 500 ng total DNA (optimized amounts of each plasmid and empty vector) and 1.5 µL Lipofectamine 2000 in 0.1 mL OptiMEM. Cells were grown overnight at 37 °C with 5% CO_2_. The next day, growth media were removed; the cells were washed with PBS, and lysed in 0.1 mL. Aliquots of lysates (5–20 µL) were added to duplicate wells of white 96-well plates. Bioluminescence was initiated by automatic injection of 0.1 ml of DSM that was either prepared fresh or thawed at room temperature after storage at −80 °C. After a 1 min delay, emission signals were recorded on a Synergy™ 2 microplate luminometer (BioTek, Winooski, VT) using 516 ± 10 nm (green) and 635 ± 16 nm (red) filters (BioTek). The instrument records the mean intensity of the flat signals over 10 s by alternately taking readings for 0.02 s with the green and red filters. To normalize the reporter signal, the mean signal over 10 s with the red filter (PLR1) was divided by the mean signal over 10 s with the green filter (PLG3).

### Validation of the DART method

To validate the DART method, we performed a previously described reporter experiment^18,19^ that employed the widely used DLR^®^ system. In HeLa^18^ and Cos-7^19^ cells, the transactivation of the MEF3/TATA promoter by Six4 and Eya2 was evaluated (Fig. 3). A set of reporter plasmids pMEF3/TATA-PLR1 and pMEF3/TATA-Luc+ was made by replacing the luc+ gene of the available^18,21^ MEF3/TATA-Luc+ vector with the cDNA of PLR1 using the *Nco*I and *Xba*I restriction sites. The internal controls were pCMV-PLG3 and the *Renilla* Luc (pRL-CMV plasmid) under the control of the CMV promoter. HEK293T cells were transfected in duplicate (as described above) with pMEF3/TATA-PLR1 (60 ng) and pCMV-PLG3 (0.5 ng) or pMEF3/TATA-Luc+ (60 ng) and pRL-CMV (10 ng). Additionally, each reporter-control pair was transfected with pcDNA3.1-Six4 (60 ng) with and without the inclusion of pcDNA3.1-Flag-Eya2 plasmid (120 ng). Twenty-four hours after transfection, the cells were washed with PBS and lysed in 0.1 mL PLB. The DART assay was performed as described above and the DLR assay was performed according to the manufacturer’s protocol^5^; 20 µL of lysate per well was used for all assays.

### Statistical analysis

Student’s *t* tests were used to assess the level of significance between methods using the Data Analysis ToolPak in Microsoft Excel.

## Electronic supplementary material

SUPPLEMENTARY INFORMATION

## References

[1] Cevenini, L., Calabretta, M. M., Calabria, D., Roda, A. & Michelini, E. In Bioluminescence: Fundamentals and Applications in Biotechnology, Vol 3, Vol. 154. (eds G. Thouand & R. Marks) 3–17 (2016).

[2] Ohmiya Y (2015). Simultaneous Multicolor Luciferase Reporter Assays for Monitoring of Multiple Genes Expressions. *Combinatorial Chem*. High Throughput Screening.

[3] Mezzanotte L, van ‘t Root M, Karatas H, Goun EA, Lowik C (2017). *In Vivo* Molecular Bioluminescence Imaging: New Tools and Applications. Trends Biotechnol..

[4] Schagat, T., Gaguio, A. & Kopish, K. Normalizing genetic reporter assays: approaches and considerations for increasing consistency and statistical significance. *Cell Notes*, 9–12 (2007).

[5] Dual-Luciferase® Reporter Assay System Technical Manual No. TM040 (Promega Corporation, 2015).

[6] Nano-Glo® Dual-Luciferase® Reporter Assay System Technical Manual No. TM426 (Promega Corporation, 2015).

[7] Kobayashi EH (2016). Nrf2 suppresses macrophage inflammatory response by blocking proinflammatory cytokine transcription. Nat. Commun..

[8] Gromadzka AM, Steckelberg AL, Singh KK, Hofmann K, Gehring NH (2016). A short conserved motif in ALYREF directs cap- and EJC-dependent assembly of export complexes on spliced mRNAs. Nucleic Acids Res..

[9] Hall MP (2012). Engineered luciferase reporter from a deep sea shrimp utilizing a novel imidazopyrazinone substrate. ACS Chem Biol.

[10] Heise K, Oppermann H, Meixensberger J, Gebhardt R, Gaunitz F (2013). Dual Luciferase Assay for Secreted Luciferases Based on Gaussia and NanoLuc. Assay Drug Dev. Technol..

[11] Ho PI (2013). Reporter Enzyme Inhibitor Study To Aid Assembly of Orthogonal Reporter Gene Assays. Acs Chemical Biology.

[12] Chroma-Glo™ Luciferase Assay System Technical Manual No. TM062 (Promega Corporation, 2015).

[13] Yasunaga M, Nakajima Y, Ohmiya Y (2014). Dual-color bioluminescence imaging assay using green- and red-emitting beetle luciferases at subcellular resolution. Anal. Bioanal. Chem..

[14] Woodroofe CC (2012). Novel Heterocyclic Analogues of Firefly Luciferin. Biochemistry.

[15] Branchini BR (2015). An enhanced chimeric firefly luciferase-inspired enzyme for ATP detection and bioluminescence reporter and imaging applications. Anal. Biochem..

[16] Branchini BR (2017). Cloning of the Orange Light-Producing Luciferase from Photinus scintillansA New Proposal on how Bioluminescence Color is Determined. Photochem. Photobiol..

[17] Branchini BR, Southworth TL, Khattak NF, Michelini E, Roda A (2005). Red- and green-emitting firefly luciferase mutants for bioluminescent reporter applications. Anal. Biochem..

[18] Fan XM (2000). The alpha subunits of G(z) and G(i) interact with the eyes absent transcription cofactor Eya2, preventing its interaction with the six class of homeodomain-containing proteins. J. Biol. Chem..

[19] Landgraf K (2010). Sipl1 and Rbck1 Are Novel Eya1-Binding Proteins with a Role in Craniofacial Development. Mol. Cell. Biol..

[20] Miraglia LJ, King FJ, Damoiseaux R (2011). Seeing the Light: Luminescent Reporter Gene Assays. *Combinatorial Chem*. High Throughput Screening.

[21] Embry AC, Glick JL, Linder ME, Casey PJ (2004). Reciprocal signaling between the transcriptional co-factor Eya2 and specific members of the G alpha i family. Mol. Pharmacol..

[22] Branchini BR (2014). A Photinus pyralis and Luciola italica Chimeric Firefly Luciferase Produces Enhanced Bioluminescence. Biochemistry.

[23] Branchini BR (2005). Mutagenesis evidence that the partial reactions of firefly bioluminescence are catalyzed by different conformations of the luciferase C-terminal domain. Biochemistry.

