# Supplementary material for: A Firefly Luciferase Dual Color Bioluminescence Reporter Assay Using Two Substrates To Simultaneously Monitor Two Gene Expression Events
Source: Sci Rep. 2018 Apr 16;8:5990. doi: 10.1038/s41598-018-24278-2 (PMC5902630; doi:10.1038/s41598-018-24278-2)
Supplement: Supplementary file 1 — SUPPLEMENTARY INFORMATION [file 41598_2018_24278_MOESM1_ESM.docx]

**SUPPLEMENTARY INFORMATION**

**A FIREFLY LUCIFERASE DUAL COLOR BIOLUMINESCENCE REPORTER ASSAY USING TWO SUBSTRATES TO SIMULTANEOUSLY MONITOR TWO GENE EXPRESSION EVENTS**

Bruce R Branchini,^1,*^ Tara L Southworth,^1^ Danielle M Fontaine,^1^ Dawn Kohrt,^2^ Catherine M Florentine,^1^ & Martha J Grossel^2^

^1^ Department of Chemistry, Connecticut College, New London, Connecticut, 06320, USA.

^2^ Department of Biology, Connecticut College, New London, Connecticut, 06320, USA.

^*^Correspondence and request for materials should be addressed to B.R.B. (brbra@conncoll.edu).

**TABLE**

| **Supplementary Table 1:**  **Bioluminescence emission maxima (nm)^a^ at pH 7.8** | | | |
| --- | --- | --- | --- |
| Enzyme | LH_2_ | BtLH_2_ | LH_2_/BtLH_2_ mix |
| PpyWT | 562 (74) | 525 (60) | 562 (74) |
| PLR1 | 620 (57) | 569 (58) | 620 (56) |
| PLG3 | 549 (74) | 518 (57) | 528 (75) |
| ^a^The full width at half-maximum is shown in parentheses. Proteins (5 μL, 1 μg) were added to 0.5 mL of assay mix containing 0.48 mM ATP-Na, 18 mM DTT, 1.8 mM MgSO_4_, and either 0.12 mM LH_2_, 0.24 mM BtLH_2_, or a mixture of 0.12 mM LH_2_ and 0.24 mM BtLH_2_. Bioluminescence spectra were taken 1 min after mixing using a Horiba Jobin-Yvon iHR imaging spectrometer equipped with a liquid N_2_ cooled CCD detector. | | | |

**SUPPLEMENTARY FIGURE 1**

**
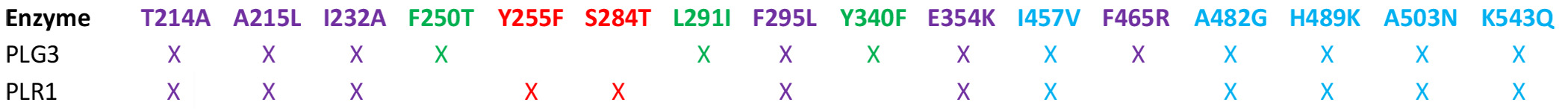
**

**Supplementary Figure 1.** Amino acid substitutions (compared with *Photinus pyralis* luciferase) in the PLG3 and PLR1 luciferase sequences. The predominant changes introduced by the substitutions are color-coded: purple, enhanced thermostability; green, increased Luc affinity ratio BtLH_2_/LH_2_; red, red-shifted bioluminescence emission; blue, increased specific activity and resistance to red-shifting at pH 6.0.

**SUPPLEMENTARY FIGURE 2**


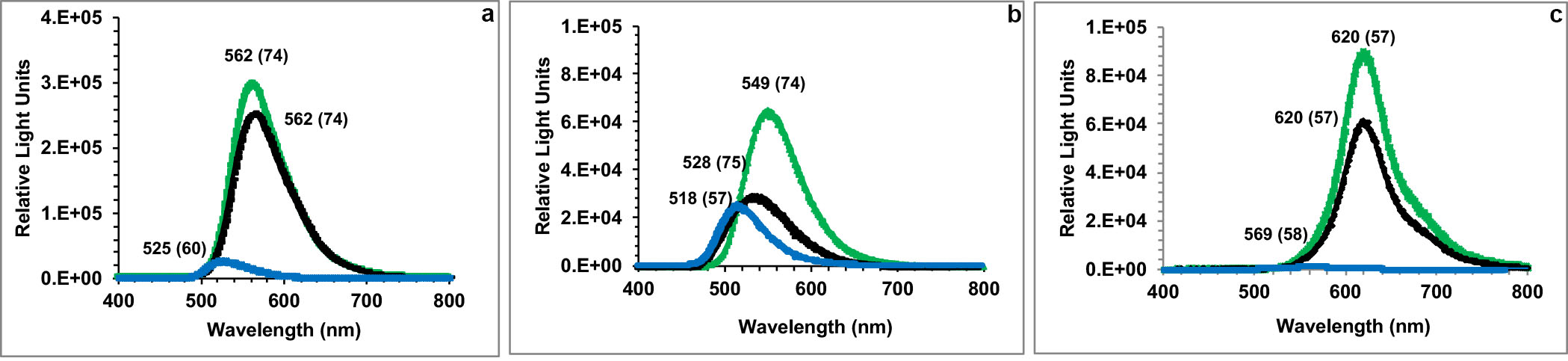


**Supplementary Figure 2.** Bioluminescence emission spectra of purified Lucs (**a**) PpyWT, (**b**) PLG3, and (**c**) PLR1 with LH_2_ (green), BtLH_2_ (blue), and a mixture of both substrates (black). The emission maxima and bandwidths are indicated above each spectrum. Proteins (1 μg) were added to 0.5 mL of assay mix containing 0.48 mM ATP-Na, 18 mM DTT, 1.8 mM MgSO_4_, and either 0.12 mM LH_2_ (green), 0.24 mM BtLH_2_ (blue), or a mixture of 0.12 mM LH_2_ and 0.24 mM BtLH_2_ (black). Bioluminescence emission spectra were measured 1 min after mixing using a Horiba Jobin-Yvon iHR imaging spectrometer equipped with a liquid N_2_ cooled CCD detector.

**SUPPLEMENTARY FIGURE 3**


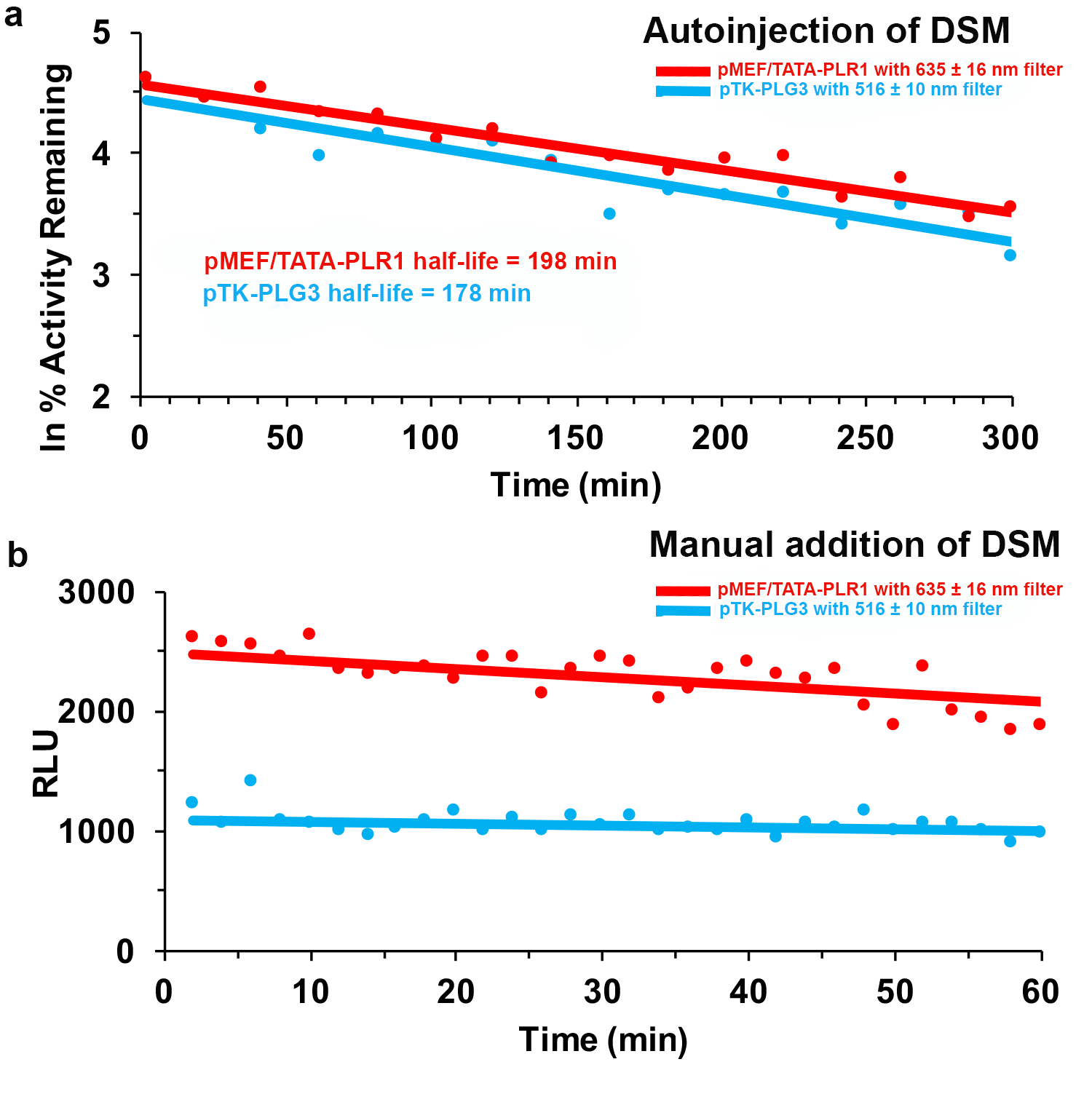


**Supplementary Figure 3.** Stability of PLG3 and PLR1 in cell lysates with DSM added manually or by automatic injection. **(a)** Semi-log plot of the % remaining Luc activity over time in lysates of equivalent numbers of HEK293T cells. Cells were dually transfected with plasmids expressing PLR1 under control of the MEF/TATA promoter (pMEF/TATA-PLR1) and PLG3 under control of the thymidine kinase promoter (pTK-PLG3). Lysates (20 μL) were added to the wells of 96-well plates and assayed using the DART method. Activity was monitored over 5 h using a Synergy™ 2 microplate luminometer equipped with the indicated filters. **(b)** Stability of DART enzymes expressed from pMEF/TATA-PLR1 and pTK-PLG3 over 1 h assayed without the use of an injector. HEK293T cells were dually transfected as described above. Lysates (20 μL) were added to the wells of 96-well plates. The DSM reagent was manually pipetted into the wells and bioluminescence was monitored 2 min after the addition of the reagent using the equipment described above.

| **SUPPLEMENTARY NOTE**  **Qualitative comparison of DART to prototypical commercial reporter systems** | | | |
| --- | --- | --- | --- |
|  | DART | Promega Dual-Luciferase Assay | Promega  Chroma-Glo |
| Number of Substrates | 2 | 2 | 1 |
| Types of Enzymes | 1 (2 FLucs) | 2 (Rluc, FLuc) | 1 (2 CBLucs) |
| Gene Identity | >99% | very low | >99% |
| Simultaneous Detection of 2 Signals | Yes | No | Yes |
| Data Calculations Required | No | No | Yes |
| Luminometer Filters Required | Yes | No | Yes |
| Single Reagent Solution | Yes | No | Yes |
| Autoinjector Required | No | No | No |
| Emission Signals Well-Separated | Yes | Yes | No |
| Background | Low | High | Low |
